# Evolution of LiNi_0.8_Mn_0.1_Co_0.1_O_2_ (NMC811) Cathodes for Li-Ion Batteries: An *In Situ* Electron Paramagnetic Resonance Study

**DOI:** 10.1021/acs.jpcc.5c00275

**Published:** 2025-04-11

**Authors:** Bin Wang, Edurne Redondo, Lewis W. Le Fevre, Adam Brookfield, Eric J. L. McInnes, Robert A. W. Dryfe

**Affiliations:** † Department of Chemistry, University of Manchester, Oxford Road, Manchester M13 9PL, U.K.; ‡ National Graphene Institute, University of Manchester, Oxford Road, Manchester M13 9PL, U.K.; § The Faraday Institution, Quad One, Harwell Science and Innovation Campus, Didcot OX11 0RA, U.K.; ∥ Photon Science Institute, University of Manchester, Oxford Road, Manchester M13 9PL, U.K.; ⊥ Henry Royce Institute, University of Manchester, Oxford Road, Manchester M13 9PL, U.K.; # Departamento de Química Orgánica e Inorgánica, Facultad de Ciencia y Tecnología, Universidad del País Vasco UPV/EHU, P.O. Box 644, Bilbao 48080, Spain

## Abstract

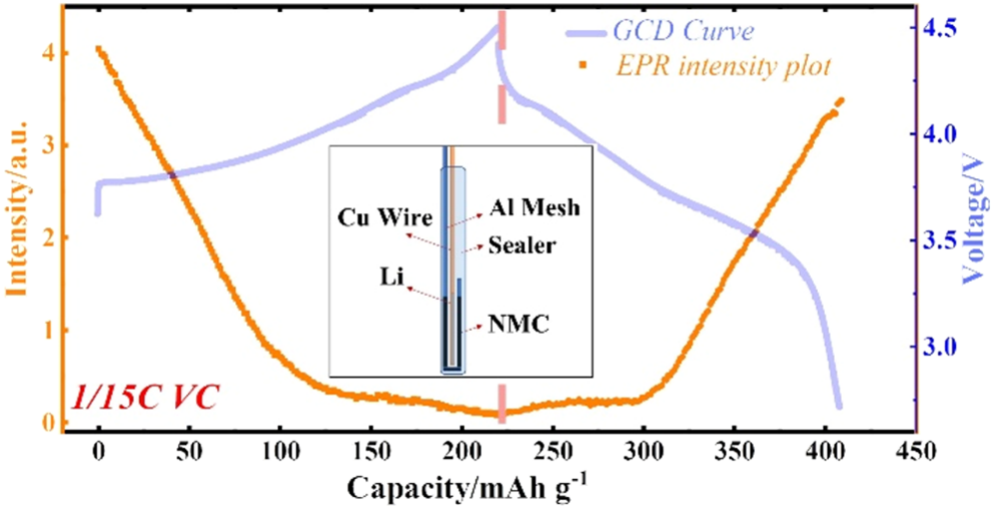

The rapid voltage and capacity fade of the otherwise promising Ni-rich layered LiNi_0.8_Mn_0.1_Co_0.1_O_2_ (NMC811) cathode are the primary obstacles to its successful commercialization in lithium-ion batteries (LIBs). Here, *in situ* electrochemical electron paramagnetic resonance (EPR) spectroscopy is employed to gain insight into the cation redox behavior of the NMC811 cathode during the cell charge/discharge process. Different oxidation states of Ni ions are detected by variations in the signal of the EPR spectra. *Ex situ* studies of NMC811 at different SOC levels also confirm changes in the local Mn–Ni environment. A comparison of *in situ* studies on fresh and cycled NMC811 electrodes demonstrates that the fundamental redox processes remain unchanged upon cycling of the material. Finally, dissolved Mn and Co ions from the bulk are found using *ex situ* EPR characterization of the cycled cathode and separator. The dissolution of these metal ions can accelerate the degradation of the entire battery.

## Introduction

Lithium-ion batteries (LIBs), known for their high power and energy density, are the preferred choice for powering portable electronic devices and electric vehicles (EVs).^1−3^ For the automotive industry, it is essential to understand the reasons and mechanisms behind capacity degradation to ensure long-term application reliability.^4^ An important aspect of enhancing the performance and durability of LIBs relies on the selection of the cathode material, where transition-metal oxides have received significant attention. Ni-rich layered LiNi_0.8_Mn_0.1_Co_0.1_O_2_ (NMC811), with a theoretical capacity of approximately 250 mAh g^–1^, is one of the most promising cathode materials. It is presented as one of the most competitive candidates to meet the current energy density target of 300 Wh kg^–1^ for EVs.^5−9^ Other advantages include its high power, its high recycle value, and its ability to operate at low temperatures. However, several challenges need to be addressed for successful commercialization, including poor chemical stability and rapid capacity loss at higher cutoff potentials (4.2 V vs. Li^+^/Li).^4,5,7,9,10^

The capacity degradation of NMC811 can be attributed to intrinsic structural changes and surface-related reactions with the electrolyte.^7,11^ Recent studies have revealed that the unit cell volume of NMC811 begins to contract at above 70% state of charge (SOC),^6,12^ leading to a significant reduction in Li mobility.^12^ Furthermore, the formation of the surface rock-salt phase (*Fm*3*m*), driven by lattice oxygen release and crystallographic defects, such as antiphase and twin boundaries,^13,14^ is believed to hinder the NMC811’s lattice expansion during delithiation (over ∼75% SOC), resulting in a fatigued layered phase during low voltage cycling (2.7–4.2 V vs. Li^+^/Li).^15^ Moreover, intragranular cracking is the primary reason for capacity fading at voltages higher than 4.2 V, which is caused by the Li concentration gradient-induced anisotropic stress and strain and the Coulombic repulsion in cation-rich regions (Ni–Li antisite defect^16^) at higher voltage ranges.^17^ The creation of these new interfaces with additional surface phase transformations increases the cell resistance and hinders the Li pathways. Additionally, the dissolution of transition-metal ions during irreversible structural fracture, which can migrate and deposit on the graphite anode, can also cause significant Li deposition.^8,18^ Finally, not only does the initial structural evolution of the NMC811 cathode affect the capacity loss,^19−21^ but these further side reactions with the electrolyte can also cause capacity loss.^11,22−25^ These reactions, associated with high Ni content, can lead to the dehydrogenation of ethylene carbonate (EC) at relatively low potentials (3.8 V vs. Li^+^/Li),^24^ potentially resulting in gas evolution and safety concerns.^25^

Electron paramagnetic resonance (EPR) demonstrates superior sensitivity (e.g., 1000-fold higher than NMR) and unique specificity toward unpaired electrons, enabling in principle the detection of all possible free radicals. Critical electrochemical energy storage processes, particularly interfacial electron transfer, may involve single- or multielectron pathways depending on the material system, and such interfacial charge transfer inevitably generates or quenches radical species. These intrinsic attributes make EPR exceptionally suited for probing electrochemical interfaces from both technical and fundamental perspectives.^26−34^ In addition to providing direct information on the valence state of metal ions through distinct spectroscopic changes (e.g., Ru^4+^/Ru^5+^, Co^2+^/Co^3+^/Co^4+^),^29,35^ EPR has also been used to investigate the cation distribution of the Li–Mn-based oxides.^36−42^ However, detecting X-band EPR spectra of lithium metal oxides containing high-spin Mn/Ni ions presents challenges due to strong spin interactions (typically the materials are magnetically concentrated), often resulting in broad single Lorentzian signals and/or large zero-field splittings depending on the metal oxidation state.^36,39,41,42^ The signal intensity and line shape are influenced by spin–spin interactions, including dipole–dipole and exchange (dependent on the distance and connectivity between spins), as well as by changes in oxidation states.^33,38,40^ For example, *operando* EPR spectroscopy of the LiNi_0.5_Mn_1.5_O_4_ spinel cathode, via line width and intensity changes, provided insights into the oxidation process, starting from residual Mn^3+^ to Mn^4+^ and further oxidation of Ni^2+^/Ni^3+^/Ni^4+^.^33^ Similar results have been obtained in the Li-rich Li_1.2_Ni_0.2_Mn_0.6_O_4_ and Li_1.2_Ni_0.13_Mn_0.5_Co_0.13_O_2_ cathodes, in which multioxidation processes of cobalt, nickel, and manganese were probed, based on changes in signal intensity over time.^32^ However, reversible O^2–^/O^2*n*–^ redox processes were not detected by EPR in these materials, as opposed to another Li-rich cathode, Li_2_Ru_0.75_Sn_0.25_O_3_.^29^

In this work, we have designed a new two-electrode *in situ* EPR cell based on a sandwich structure. This cell includes a metallic Li anode and a free-standing NMC811 film cathode, allowing us to study the redox reactions of metal cations at room temperature under galvanostatic cycling. We employed this approach to assess the multiple oxidation stages of Ni ions by monitoring the signal intensity changes, and we correlated these data with electrochemical measurements performed on the same cell. Additionally, we report changes in the local Mn–Ni environment in NMC811 during electrochemical extraction and reinsertion of lithium: these are investigated through further *ex situ* EPR experiments at lower temperatures. Finally, we resolved the capacity degradation and transition-metal dissolution through *in situ* and *ex situ* EPR characterization of cycled cathodes under various conditions.

## Experimental Section

### In Situ EPR Cell Fabrication

**Figure 1:**
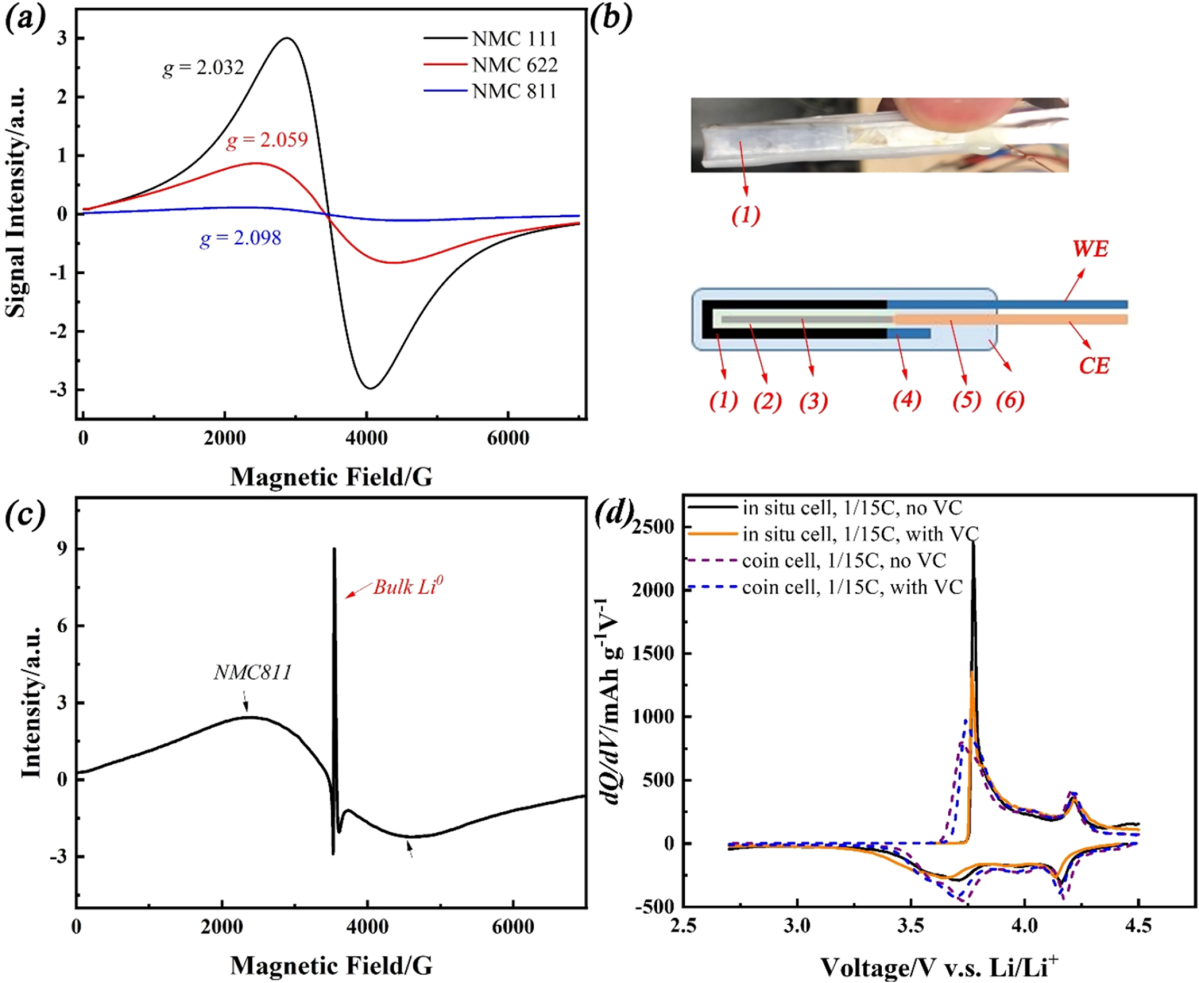
(a) EPR spectra comparing NMC-type cathode powder samples, with the mass normalized to 1 mg, at room temperature. (b) Photograph (above) and scheme (below) of the sandwich structure *in situ* EPR cell: (1) NMC811 free-standing film (thickness ∼ 75 μm) compressed onto conductive Al mesh as the working electrode (WE); (2) separator (Celgard^@^ 2325, thickness 25 μm) coated onto the Li, preventing short circuit between the anode and the cathode; (3) Li film coated onto the Cu wire as counter electrode (CE); (4) Al mesh and (5) Cu wire covered with a layer of insulating polymer as the current collector; (6) plastic bag to seal the prepared two-electrode cell. (c) EPR spectra of the *in situ* cell at room temperature. (d) Comparison of d*Q*/d*V vs* voltage plots between the *in situ* cell and the coin cell of the NMC811 free-standing cathode.

NMC811 cathode was made by grinding, rolling, and folding 90 wt % NMC811 (Targray), 5 wt % Ketjen black (Timcal C45), and 5 wt % powder poly(tetrafluoroethylene) (PTFE) (Sigma-Aldrich) until a shiny, flexible film with the thickness around 75 μm was obtained. Then, a piece of NMC free-standing film (∼0.25 cm × 15 cm, mass loading around 15 mg) was compressed onto the Al mesh (Advent Research Materials Ltd., U.K.) for the working electrode. The prepared working electrode was covered with a layer of separator (Celgard^@^ 2325, thickness 25 μm) before drying in the vacuum oven at 120 °C overnight. The reference/counter electrode was made in the argon glovebox by rolling a layer of Li onto the Cu wire (insulated, diameter 0.5 mm, Advent Research Materials Ltd., U.K.). The in situ cell was assembled in the glovebox (O_2_ ≤ 5 ppm; H_2_O ≤ 1 ppm) by putting the counter electrode (Li) into the middle of the NMC working electrode as shown in Figure 1. The advantage of a sandwich geometry for the in situ cell is the reduction of the EPR signal due to metallic Li, which is much stronger than the weak NMC811 signal. The microwaves cannot pass through the NMC811 film (assuming a skin depth of 1.1 μm at a microwave frequency of 9.8 GHz), and the detected EPR signal of the Li counter electrode should be very weak. The two electrodes were twined with PTFE tape (EPR silent) to reduce the connection resistance. Then, the prepared two-electrode cell was immersed into the electrolyte (LP57:1 M LiPF_6_ in 3:7 ethylene carbonate/ethyl methyl carbonate (EC/EMC), with/without 2% vinylene carbonate (VC) additive). The surface-coated electrolyte was removed before sealing it into the plastic bag for further EPR measurement.

The *in situ* EPR experiment was also carried out on the cycled NMC811 cathode. The NMC811 free-standing film was first assembled into a coin cell by using the Li as the counter electrode for a further 100 cycles at 1/3C in LP57 with/without the VC additive over a voltage range from 2.7 to 4.2 V. Then, the cycled NMC811 was removed from the coin cell and assembled into the *in situ* EPR cell.

### *In Situ* EPR Measurement

The EPR spectra were recorded at room temperature using a continuous-wave (CW) Bruker EMX Micro spectrometer, with a microwave frequency of 9.8 GHz, a modulation amplitude of 10 G, a sweep width of 7000 Gs, a microwave power of 6.325 mW, and a sweep time of 30.03s. The spectra were the average of two scans. The electrochemical process was measured in galvanostatic mode by an Autolab electrochemical workstation (Metrohm, model PGSTAT 302N) under 1/10 and 1/15 C from 2.7 to 4.5 V.

For the *in situ* EPR characterization of the cycled NMC811, the free-standing NMC electrode is first cut to an appropriate size and then cycled under different conditions in a coin cell. The cycling conditions are as follows: 1/3 C for 100 cycles from 2.7 to 4.2 V in LP57 electrolyte, with and without VC additive, tested in a coin cell. After cycling, the coin cell is transferred into a glovebox, where it is opened to retrieve the cycled NMC811 film. This electrode is then further assembled into an in situ EPR cell for further measurement.

*Ex situ* EPR measurement: the free-standing NMC811 was charged/discharged under the C rate of 1/20 to a specified voltage in a coin cell, e.g., charging to 4 V and discharging to 2.7 V. The cell was opened in the glovebox, and the NMC811 electrode was taken out and washed with EMC twice by slowly flowing over the surface. After drying in the glovebox, the NMC811 electrode was sealed into the EPR tube for further measurement at liquid helium temperature. The microwave frequency was 9.4 GHz, the modulation amplitude was 4 G, the sweep width was 7000 Gs, the microwave power was 2.193 mW, and the sweep time was 70.35s. The spectra were the average of 5 scans. The spin susceptibility is simulated by the Curie–Weiss function: χ = *C*/(*T* – *T*_C–W_); χ is the susceptibility calculated from the double integration value of measured EPR spectra, *C* is the Curie constant, *T* is the measured temperature (K), and *T*_C–W_ is the Curie–Weiss temperature.

## Results and Discussion

NMC materials contain multiple metal ions, including paramagnetic Ni^2+^, Ni^3+^, Mn^4+^, Co^4+^, and diamagnetic Co^3+^.^43^ The magnetic properties of three different NMC layered oxides were investigated to gather some background information. Figure 1a shows the EPR spectra of LiNi_1/3_^2+^Mn_1/3_^4+^Co_1/3_^3+^O_2_ (NMC111), LiNi_0.2_^2+^Ni_0.4_^3+^Mn_0.2_^4+^Co_0.2_^3+^O_2_ (NMC622), and LiNi_0.1_^2+^Ni_0.7_^3+^Mn_0.2_^4+^Co_0.2_^3+^O_2_ (NMC811) in powder form.^43^ The three samples show a single broad resonance each. The signal intensity (normalized per mass) increases as the stoichiometry of Mn increases in the NMC-type materials; hence, the Mn^4+^ (*S* = 3/2) content (and its interactions with other ions) appears to dominate the EPR spectra. This has been concluded previously for NMC811 and other NMC and Ni/Mn oxides such as LiNi_0.5_Mn_1.5_O_4_^33,42^ and has been attributed to the very broad spectra of the non-Kramers Ni^2+^ ion (*S* = 1) cf. the Kramers Mn^4+^ ion.^44^ Progressive replacement of the low-spin and diamagnetic Co^3+^ (*S* = 0) by paramagnetic Ni^2+^ and Ni^3+^ (*S* = 1/2) leads to a positive shift of the *g*-value and a broadening of the line width due to additional spin–spin interactions with the paramagnetic Mn^4+^ ions.^39,42^ Thus, NMC111 shows a *g*-value of 2.032 and a line width of 198 mT, whereas NMC811 has a *g*-value of 2.098 and a line width of 230 mT. These compare with a line width of ca. 100 mT found in LiNi_0.5_Mn_1.5_O,^33^ again highlighting the effect of the Ni content. In this work, we have focused on the study of NMC811 because Ni-rich cathodes tend to increase the energy density and improve overall battery performance. Furthermore, it has a higher capacity and a lower Co content, which is a critical material and a cause for environmental concern relating to current mining practices.^45^Figure S1a shows the EPR spectrum of the solid-state NMC811, which is fitted to a single Lorentzian line shape. Fitting the signal intensity as a function of temperature to the Curie–Weiss equation gives a Weiss temperature of −14 K, implying dominant antiferromagnetic interactions between cations, and in good agreement with the result from Dutton and co-workers from magnetometry data.^46^

For the electrochemical analysis of the NMC811 cathode material, a new sandwich *in situ* EPR cell has been designed, which is based on a two-electrode cell system in a plastic bag (length 20 mm, width 2–2.5 mm, as shown in Figure 1b), which is sealed in a glovebox. A detailed description of the cell fabrication is given in the Experimental Section. This *in situ* cell, which contained both an NMC811 cathode and a Li counter/reference electrode, was placed inside the EPR resonator for the experiments. A sandwich design was adopted, covering the bulk Li with the cathode material. This partially dampens the EPR signal due to bulk Li because of the microwave absorption skin depth effect. Figure 1c shows the EPR spectrum of the *in situ* cell on initial set up at the open circuit voltage (OCV). Two signals can be distinguished: one narrow resonance from both metallic Li and the conductive carbon due to their similar *g*-value (close to 2.003) and one broad resonance from the NMC811 cathode material. The *in situ* cell was also tested for its electrochemical response. Figure S2 shows the galvanostatic charge–discharge cycles of the *in situ* cell and the coin cell. When tested in the *in situ* EPR cell, NMC811 shows a capacity value of 193 mAh g^–1^ at 1/15 C when charged to 4.5 V in 1 M LiPF_6_ in 3:7 ethylene carbonate/ethyl methyl carbonate (EC/EMC, LP57) without the vinylene carbonate (VC) additive. This value is lower than the one obtained when it was tested in a coin cell (225 mAh g^–1^) under the same conditions. This decrease in capacity can be related to a higher resistance of the *in situ* cell due to the limited internal pressure and the use of the limited amount of electrolyte to minimize nonresonant microwave absorption. d*Q*/d*V vs* voltage plots of the *in situ* cell and the coin cell are given in Figure 1d. The two types of cells show comparable results, with three main peaks appearing at 3.7, 4.0, and 4.2 V (vs Li/Li^+^), in agreement with the previous literature.^12,19^ These peaks have been ascribed to phase transitions of the NMC811: (i) from hexagonal to monoclinic H1 → M, (ii) from monoclinic to hexagonal M → H2, and (iii) from hexagonal to hexagonal H2 → H3.^47^ In summary, the newly developed sandwich-style *in situ* EPR cell enables the study of cathodic redox reactions under realistic cycling conditions with an electrochemical response similar to a coin cell format.

**Figure 2:**
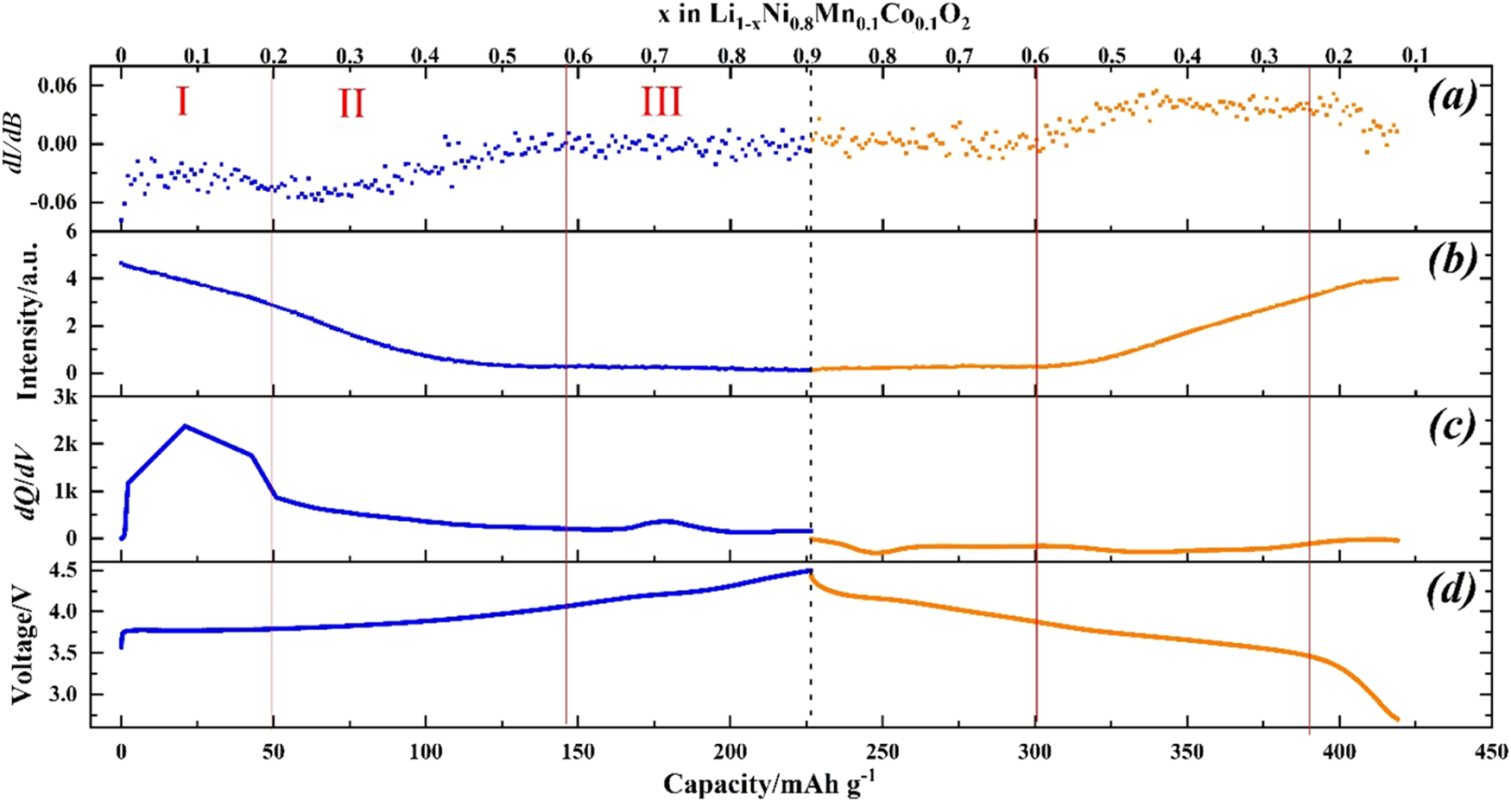
(a) First derivative of (b) the EPR signal intensity of NMC811 (broad signal); (c) d*Q*/d*V* and (d) the voltage change of the NMC811 cathode during charge (blue) and the discharge (orange) process in LP57 without VC additive at a C rate of 1/15C from 2.7 to 4.5 V during its first cycle.

Using the *in situ* EPR cell, an *operando* study of the NMC811 cathode material was carried out. EPR spectra were collected approximately every 4 min while the applied current was kept constant at 1/15 C (about 0.2 mA), starting from the OCV, at around 3.5 to 4.5 V vs. Li/Li^+^ in LP57, as shown in Figure S3. The narrow signal at around 3500 G due to the exposed metallic Li (see above) increased and decreased over the charging and discharging processes, respectively, as lithium is plated and dissolved.^48^ Achieving a quantitative analysis of Li deposition and stripping is difficult as the particle size can influence the response of the signal intensity.^34^ It is also possible that oxygen functional groups in conductive carbon materials contribute to signal changes (slight shift of the *g*-value and signal intensity) during charge–discharge processes; however, these subtle variations are obscured by the metallic lithium signal. The broad signal, due to the NMC811, continuously decreased when sweeping to a higher working electrode voltage (as summarized in Figure 2). The signal intensity decreased quickly in regions I and II and slowed when the cell was further charged in region III. The EPR signal change is reversible on decreasing the voltage from 4.5 to 2.7 V (Figure S3c,d) when the cell is discharged. No additional signals were found at room temperature, suggesting a homogeneous redox reaction process. Specifically, there is subtle broadening on charging as the signal decreases at room temperature, similar to what was found for the LiNi_0.5_Mn_1.5_O_4_ cathode.^33^

Figure 2 summarizes the *in situ* EPR data with the NMC811 cathode in LP57 at a 1/15C rate. The EPR signal intensity and its first derivative have been correlated with the voltage, and d*Q*/d*V* has been calculated from the electrochemical measurements on the same cell. As shown earlier, the intensity of the broad EPR signal decreased as the cell was charged. This is consistent with the oxidation of paramagnetic Ni^2+^ and Ni^3+^ ions to diamagnetic Ni^4+^ ions, consistent with previous work on related materials,^32,33^ as supported via spectroscopic studies.^49−51^Figure 2a suggests that three different regions of the delithiation of NMC811 exist based on the changes in the derivative of the signal intensity as a function of Li content, and these regions can be related to the different valence state changes of Ni ions and to the Li dynamics. Region I, from *x* = 0 in Li_1–*x*_Ni_0.8_Mn_0.1_Co_0.1_O_2_ to *x* = 0.2, and region II, from *x* = 0.2 to 0.6, show negative slopes of the EPR signal, as similarly found for Li_1.2_Ni_0.13_Mn_0.54_Co_0.13_O_2_^32^ and LiNi_0.5_Mn_1.5_O_4_.^33^ These are related to the oxidation of Ni^2+^ to Ni^3+^ and further to Ni^4+^. Depleting the paramagnetic content causes a decrease in the signal, and when all Ni completes its oxidation, the slope of the EPR signal levels off. Further oxidation of NMC811 (region III, *x* ≥ 0.6) shows almost no change in the intensity of the EPR signal, consistent with no further changes in metal ion oxidation states. The mechanism involved in this latter region has been the focus of many studies: some of them suggest that anionic redox reactions of oxygen (O^2–^ → O_2_^*n*–^) are involved, and others have found that severe parasitic reactions occur between the electrode and the electrolyte at this stage.^52−55^

**Figure 3:**
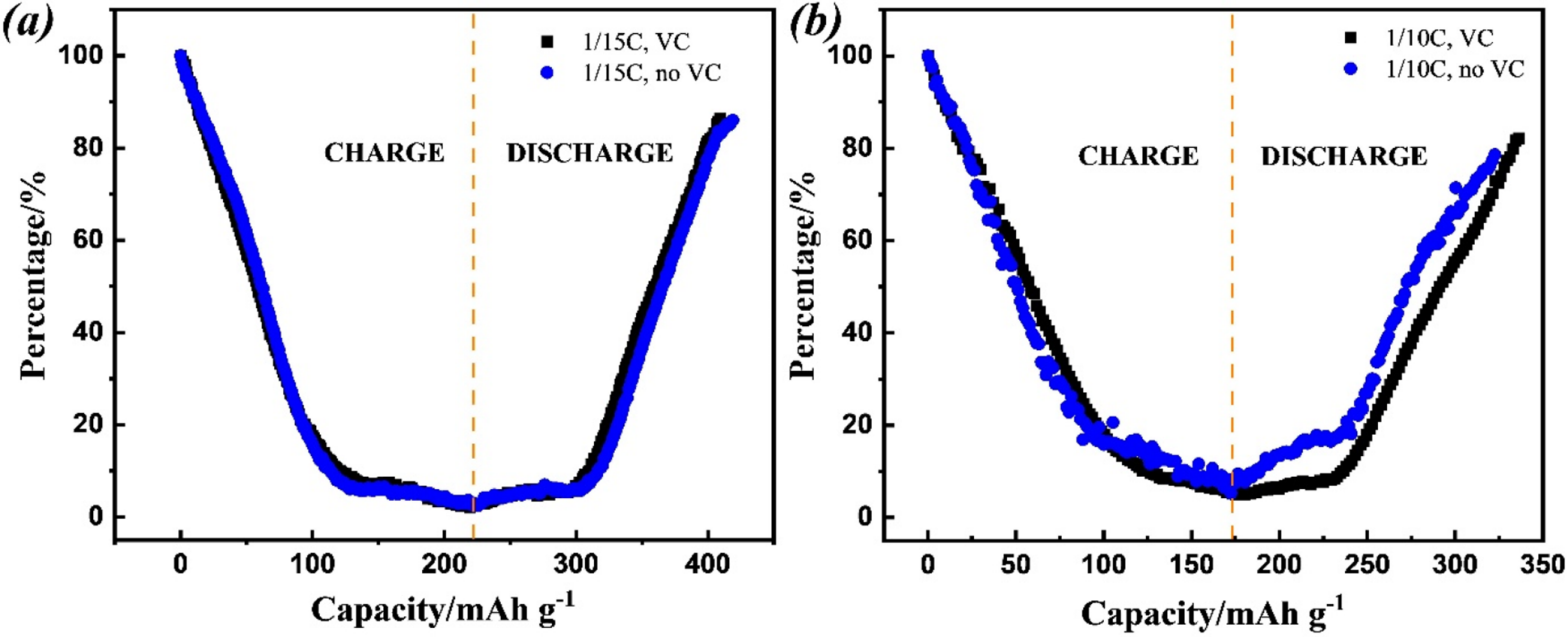
Normalized EPR signal intensity of NMC811 under different SOC in LP57 with and without VC additive at a C rate of (a) 1/15C and (b) 1/10C from 2.7 to 4.5 V during its first cycle.

Equivalent experiments were carried out using vinylene carbonate (VC) as an additive in LP57 and using different cycling conditions, as VC is known for its positive effect on capacity retention.^19,56,57^ The results obtained from the initial first cycles under different C rates (1/10 or 1/15 C) are summarized in Figures 3 and S4–S6. For example, the evolution of the signal attributed to the delithiation of NMC811 in the presence of the VC additive at 1/15C is shown in Figure S4. The results were similar to those presented above for the cell without VC additive. Three similar regions can be identified, both for cell charge and discharge, with a similar EPR response, suggesting that the addition of the VC does not significantly influence the redox processes in the first cycle. However, the addition of VC played a positive role in improving the cyclic stability of the material, as characterized by the significant differences observed in subsequent EPR spectra (*vide infra*). Additionally, while the performance of the *in situ* cell may exhibit some deterioration at higher C rates compared to coin cells, the similarity in alterations observed in the EPR spectra provides evidence for the reversibility of the overall process.

**Figure 4:**
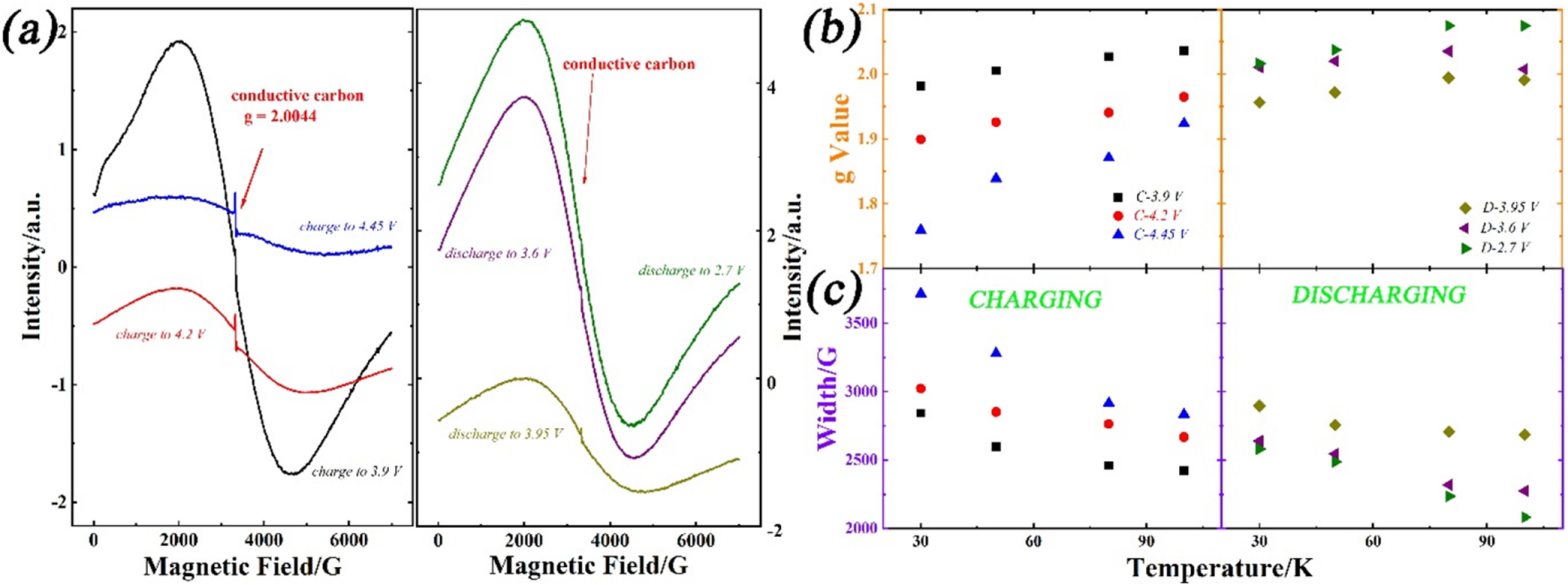
Summary of the *ex situ* EPR results of NMC811 cathodes under various conditions when the coin cell was operated under 1/15 C in LP57 without the VC additive. (a) EPR spectra of NMC811 cathode at 50 K; (b) the *g*-value; and (c) the line width of NMC811 under various temperatures. (■) sample charged to 3.9 V; (red circle solid) sample charged to 4.2 V; (blue triangle up solid) sample charged to 4.45 V; (yellow diamond solid) sample discharged to 3.95 V after charging to 4.2 V; (purple triangle left-pointing solid) sample discharged to 3.6 V after charging to 4.2 V; (green triangle right-pointing solid) sample discharged to 2.7 V after charging to 4.2 V.

Further *ex situ* EPR studies were conducted at the X-band at lower temperatures (20–100 K). For this, coin cells were operated under various conditions and the electrodes were extracted for their analysis. Figure 4 shows the EPR spectra of the extracted electrodes. The EPR signal of NMC811 under different SOCs shows a similar broad resonance. In addition, a narrow EPR signal centered around 3500 G, with a *g*-value of 2.0044, appeared during the charging process. This newly generated EPR signal could be related to the conductive carbon additive and its oxidation to yield oxygen-containing functional groups (such as carboxylates or quinones).^58,59^Figure 4b,c summarize the temperature dependence of the line width and the *g*-value of the broad signal from NMC811 under various SOC. At lower temperatures, the broadening of the line width, along with a negative shift in the *g*-value on charging the cathode, is observed and is more apparent than at room temperature. The changes are reversible during the discharge process. The broadening on charging is consistent with the literature on other Ni/Mn oxides^33,39,42^ and has been attributed to the decreased number of Ni^2+^ ions in the first metal coordination sphere of Mn^4+^, reducing exchange-narrowing interactions with Mn^4+^.

**Figure 5:**
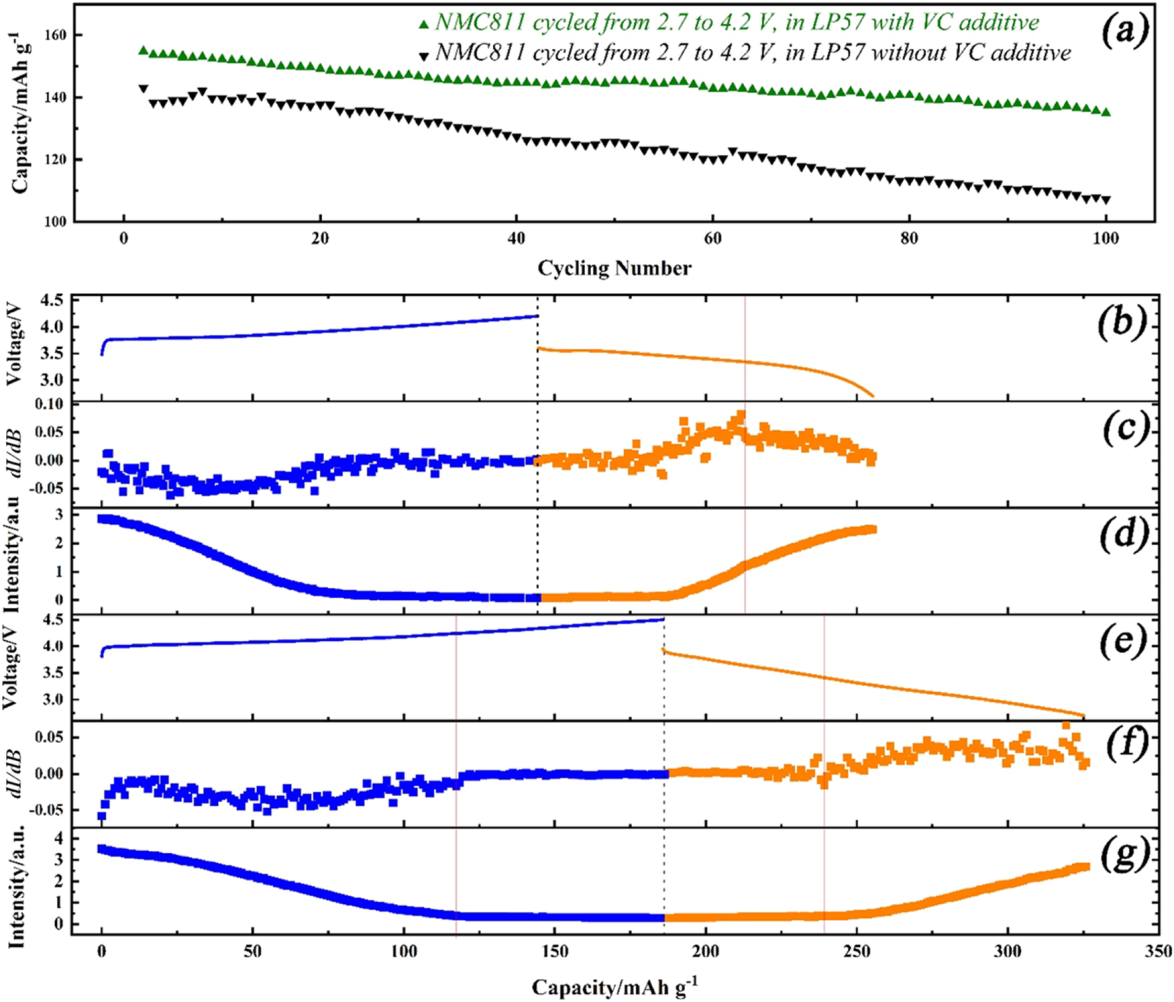
(a) Cycling capability of NMC811 electrodes at 1/3 C for 100 cycles from 2.7 to 4.2 V in LP57 electrolyte with and without VC additive tested in coin cell. Summary of the *in situ* EPR results of the NMC811 electrode after 100 cycles in LP57 from 2.7 to 4.2 V: (b) the voltage change during charge (blue) and the discharge (orange) process, (c) the first derivative of the EPR signal intensity, and (d) the EPR intensity vs capacity. *In situ* EPR results of the NMC811 electrode after 100 cycles in LP57 with 2% VC from 2.7 to 4.2 V: (e) the voltage change, (f) the first derivative of the EPR signal intensity, and (g) the EPR intensity vs capacity.

Cycling tests of NMC811 were carried out on the coin cell, from 2.7 to 4.2 V, after activation at 1/15 C for two cycles, using the LP57 electrolyte with and without VC additive. Figure 5a shows the results from the cyclability test. Without VC, there is a capacity decay at the highest cutoff voltage (i.e., 4.2 V), which can be attributed to the formation of inactive phases. This is primarily caused by the presence of surface-reconstructed rock-salt layers and the degradation of the electrolyte, a consequence of the high nickel content on the surface.^15^ The improved cycling properties with the use of VC additive indicate a significant inhibitory effect on rock-salt surface layer formation.^14,19,21^ This aligns with the lower IR drop observed in the *in situ* EPR cell when the cathode was cycled with the VC additive, as presented in the charge/discharge curve in Figure 5b,e. The evolution of the EPR signal intensity, attributed to the oxidation of Ni ions, was similar to the results presented above (Figure 2). However, the EPR data cannot distinguish either the structural transformation (e.g., rock-salt surface layer) or the inactive phase.

**Figure 6:**
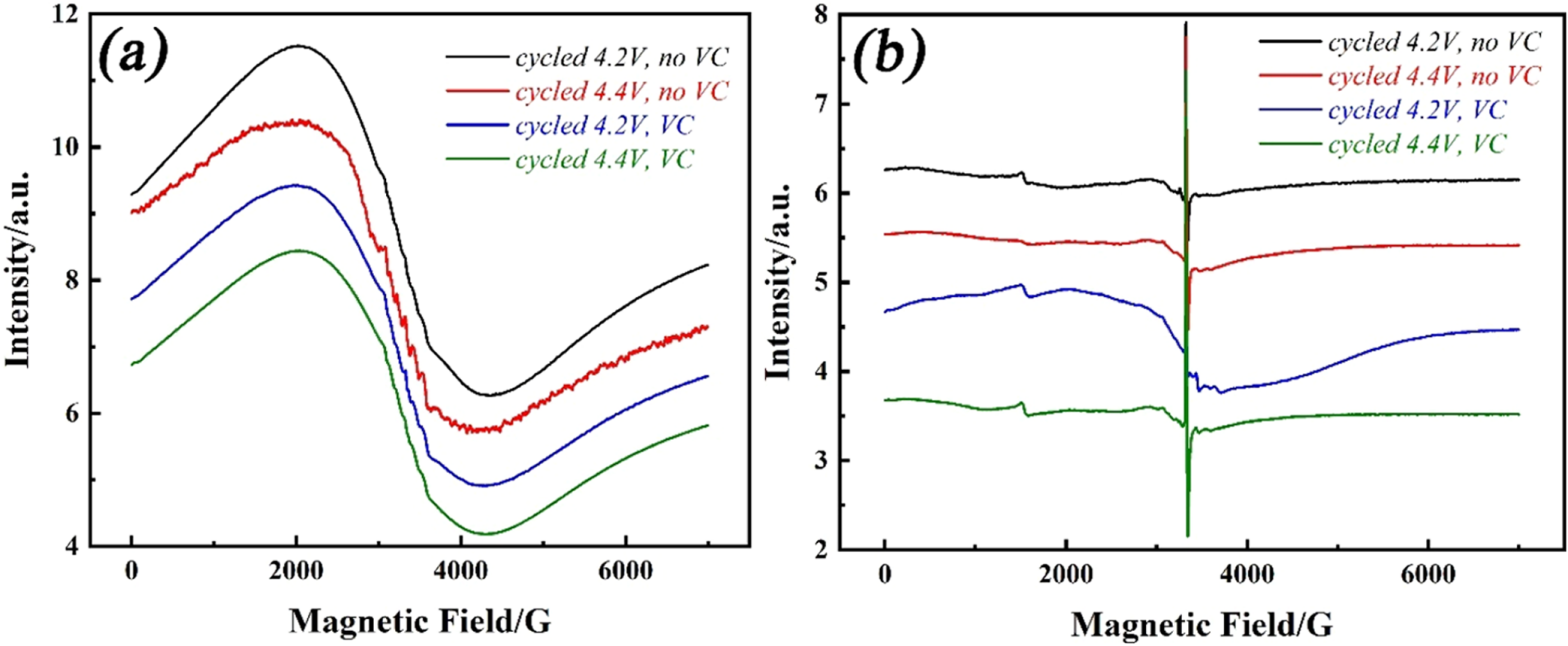
EPR spectra at 50 K of (a) NMC811 and (b) the separator from a two-electrode coin cell cycled at 1/3 C for 100 cycles from 2.7 V to 4.2 or 4.4 V with or without 2% VC additive.

Further *ex situ* low-temperature EPR characterization of the cycled cathodes and separators was carried out to gather additional insight into the degradation of the electrodes. Figure 6a shows the EPR spectra of the cycled NMC811 electrodes and separator after 100 cycles. All of the cycled NMC811 cathodes, including the control experiment with VC additive under 4.2 and 4.4 V cutoff voltages, exhibited broad Lorentzian resonance signals similar to those described above. However, there is a narrow hyperfine sextet structure centered on *g* = 2.00, characteristic of Mn^2+^ (*S* = 5/2) in all of the samples. This suggests that metal ion dissolution from the bulk material has occurred, as widely reported via other methods.^60−62^ Measurements of the separator after cycling (Figure 6b) yield more information about metal ion dissolution. There is a sharp signal centered at a *g*-value of 2.001, which is attributed to the conductive carbon attached to the separator. In addition to the six-line hyperfine structure of Mn^2+^, as for the cycled cathodes, a weak high-spin Co^2+^ (*S* = 3/2) EPR response was also found at an effective *g*-value of 4.27.^63^ The presence of Co^2+^ can be associated with the electrochemical reduction of the metal ions, and it can be explained by the following reaction: MO_2_ → M^2+^ + 2^e–^ + O_2_.^61^ The detection of reduced metal ion signals (Mn^2+^ and Co^2+^) confirms that they can be dissolved from the bulk material during cycling, consistent with recent findings.^61^ We did not detect any signal from Ni^2+^; however, such ions can be EPR silent at these microwave frequencies due to large zero-field splitting and spectral broadening.^64^

## Conclusions

A two-electrode *in situ* EPR cell, designed in a sandwich-like configuration, has been constructed to investigate the redox reactions of cathode materials under constant current density at room temperature. By integrating the fabrication process of free-standing electrodes, we can achieve *in situ* EPR testing of the electrodes after cycling in a coin cell. The early stage capacity increase (Region I, SOC ≤ 15%) results from the oxidation of Ni^2+^ to Ni^3+^. Subsequent capacity increases (Regions II and III) are attributed to continuous oxidation to Ni^4+^. Changes in Li dynamics due to structural evolution at higher SOC (≥0.6, Region III) lead to a slower oxidation process, as indicated by a subtle change in the signal intensity slope. *Ex situ* EPR of NMC811 at various SOC levels reveals line width broadening during delithiation, which can be attributed to reduced spin interactions between Ni and Mn. The addition of VC exhibits a pronounced inhibitory effect on the cathode degradation during extended cycling. In contrast, *in situ* EPR of cycled NMC811 cathodes demonstrates that the fundamental redox process remains unchanged, and the capacity loss at a cutoff voltage of 4.2 V is most likely related to the presence of inactive phases within the bulk materials. The dissolution of metal ions, such as Co and Mn, is confirmed through *ex situ* EPR spectra of both the cycled cathodes and the separators.

## Supporting Information Available

EPR simulation of the NMC811 and its spin susceptibility, GCD curves of the in situ/coin cell, in situ EPR signal change of the NMC811 during its first cycle, and in situ EPR results summarized under various conditions (PDF)
