# Supplementary material for: Evolution of LiNi0.8Mn0.1Co0.1O2 (NMC811) Cathodes for Li-Ion Batteries: An In Situ Electron Paramagnetic Resonance Study
Source: J Phys Chem C Nanomater Interfaces. 2025 Apr 11;129(16):7667–76. doi: 10.1021/acs.jpcc.5c00275 (PMC12035850; doi:10.1021/acs.jpcc.5c00275)
Supplement: Supplementary file 1 — jp5c00275_si_001.pdf [file jp5c00275_si_001.pdf]

Supporting Information:

Evolution of $LiNi_{0.8}Mn_{0.1}Co_{0.1}O_2$ (NMC811) Cathodes for Li-ion Batteries: an *in situ* Electron Paramagnetic Resonance Study

Bin Wang[a,b,c#], Edurne Redondo[a,d,e,f], Lewis W. Le Fevre[a,b,e], Adam Brookfield[a,d], Eric J. L. McInnes[a,c,d]* and Robert A. W. Dryfe[a,c,e]*

a = Department of Chemistry, University of Manchester, Oxford Road, Manchester M13 9PL, U.K.;

b = National Graphene Institute, University of Manchester, Oxford Road, Manchester M13 9PL, U.K.;

c = The Faraday Institution, Quad One, Harwell Science and Innovation Campus, Didcot, OX11 0RA, U.K;

d = Photon Science Institute, University of Manchester, Oxford Road, Manchester M13 9PL, U.K.;

e = Henry Royce Institute, University of Manchester, Oxford Road, Manchester M13 9PL, U.K.;

f = Departamento de Química Orgánica e Inorgánica, Facultad de Ciencia y Tecnología, Universidad del País Vasco UPV/EHU, P.O. Box 644, 48080 Bilbao, Spain.

Corresponding author emails:

eric.mcinnes@manchester.ac.uk; robert.dryfe@manchester.ac.uk

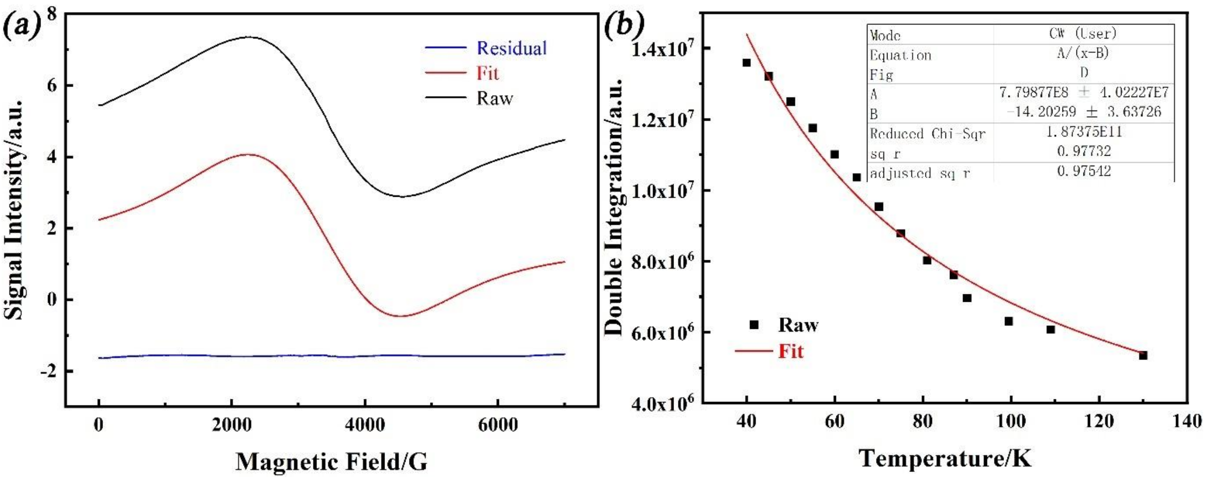

Figure S1. (a) NMC811 lineshape simulation with single Lorentzian signal; (b) spin susceptibility and its Curie-Weiss simulation result.

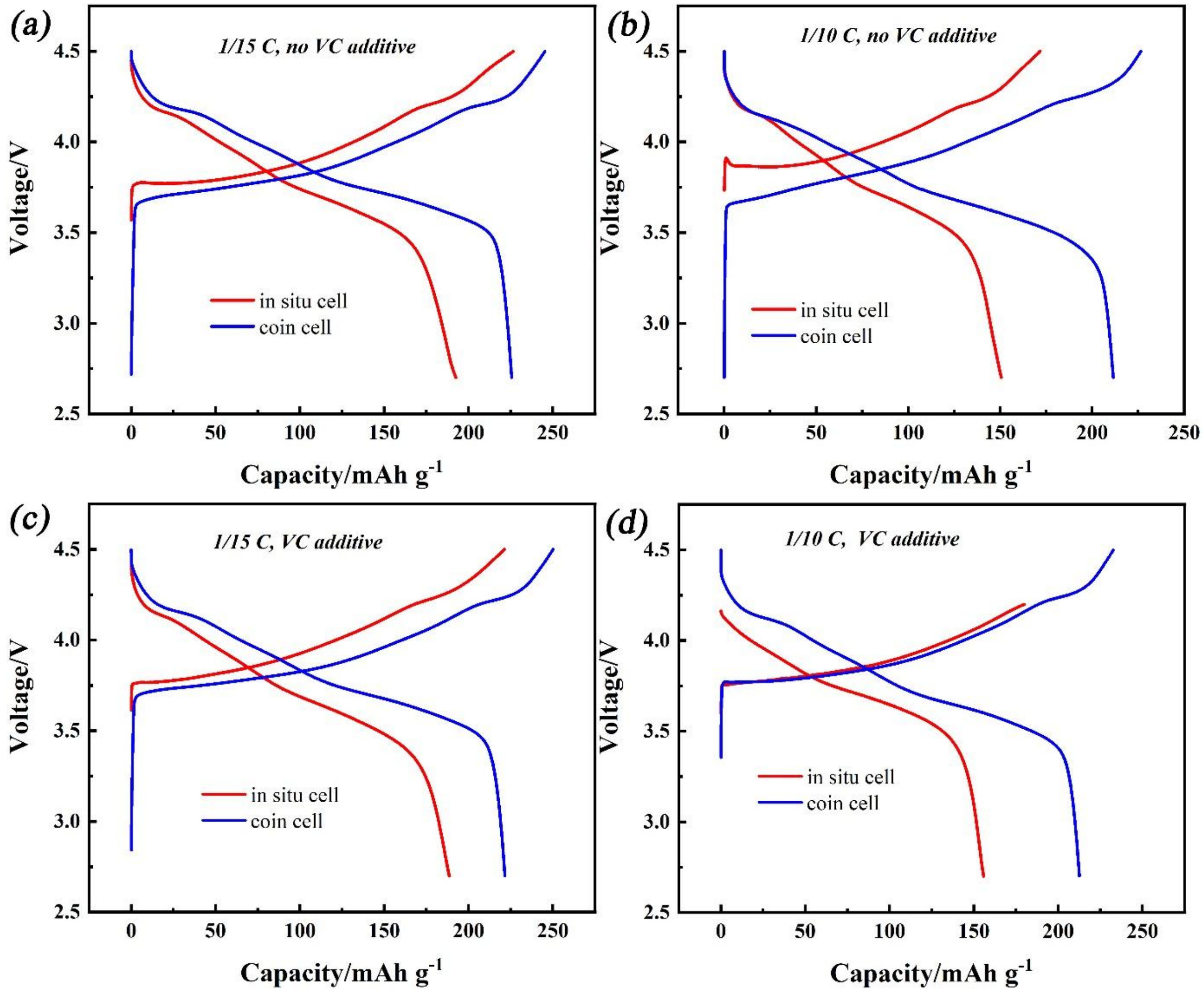

Figure S2. Electrochemical comparison between the fabricated two -electrode *in situ* EPR cell and the corresponding coin cell in LP57 with and without the VC additive at 1/10 and 1/15C rate from 2.7 V to 4.5 V. In Figure (d), the voltage window of the *in situ* cell extends from 2.7 to 4.2 V.

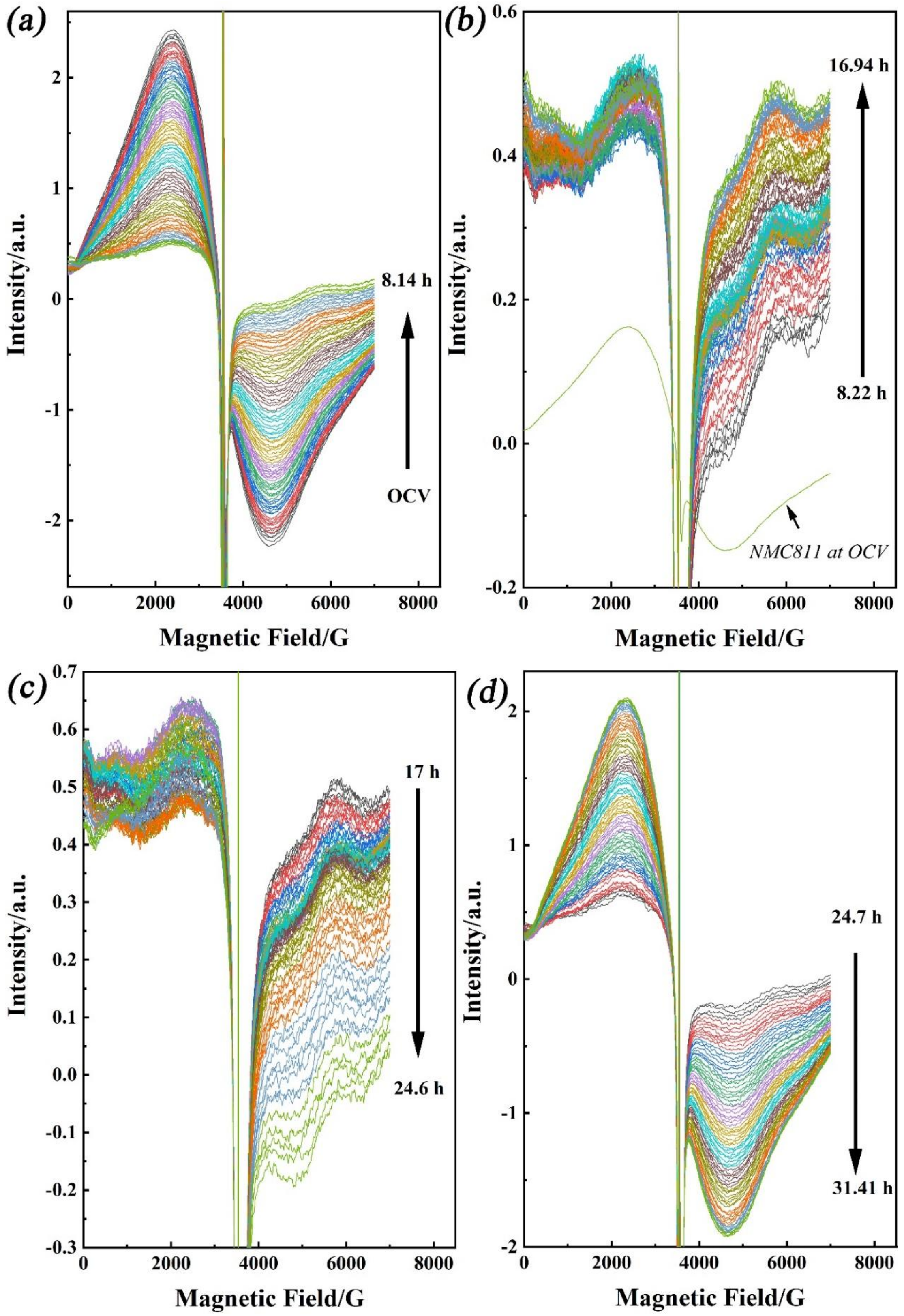

Figure S3. *In situ* EPR spectra of the NMC811 cathode: (a and b) during charging and (c and d) during discharging, at a C rate of 1/15C from 2.7 V to 4.5 V, in LP57 without 2% VC additive during its first cycle.

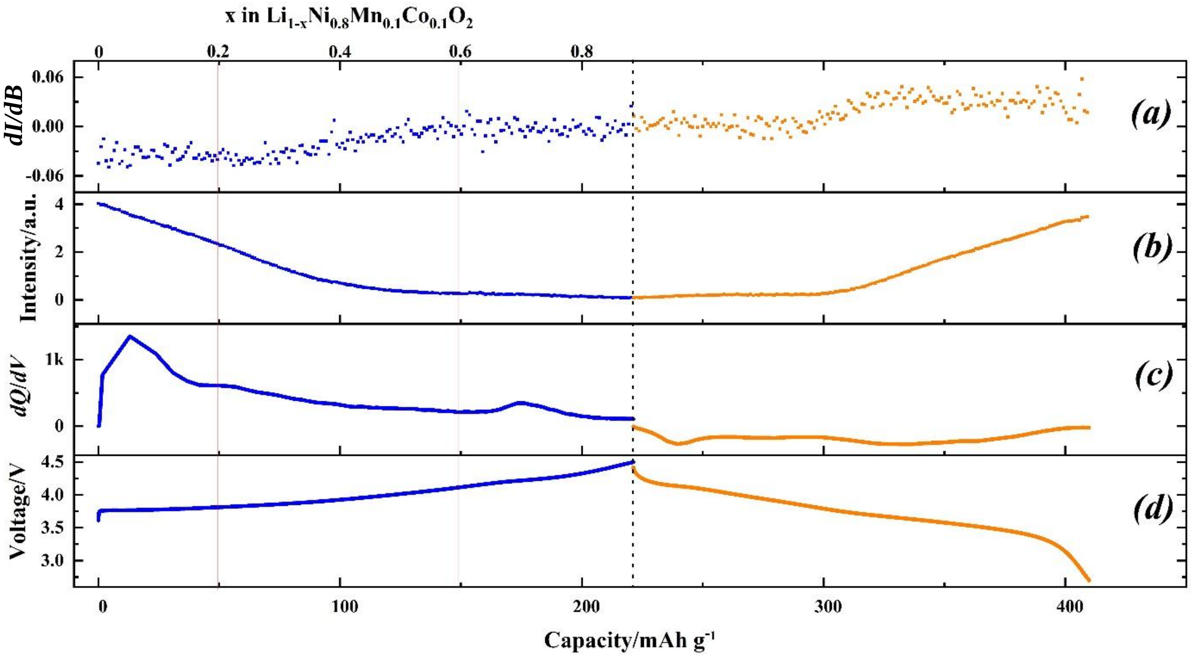

Figure S4. (a) The first derivative of (b) the EPR signal intensity of NMC811 (broad signal); (c) *dQ*/*dV* and (d) the voltage change of the NMC811 cathode during charge (blue) and the discharge (orange) process in LP57 with 2% VC additive at a C rate of 1/15C from 2.7 V to 4.5V during its first cycle.

The x($Li_{1-x}Ni_{0.8}Mn_{0.1}Co_{0.1}O_2$) is calculated from the theoretical capacity of NMC811: (mAh/g) = [26800/Mol.wt]×No. of exchangeable Li, where x denotes the tested capacity/theoretical capacity.

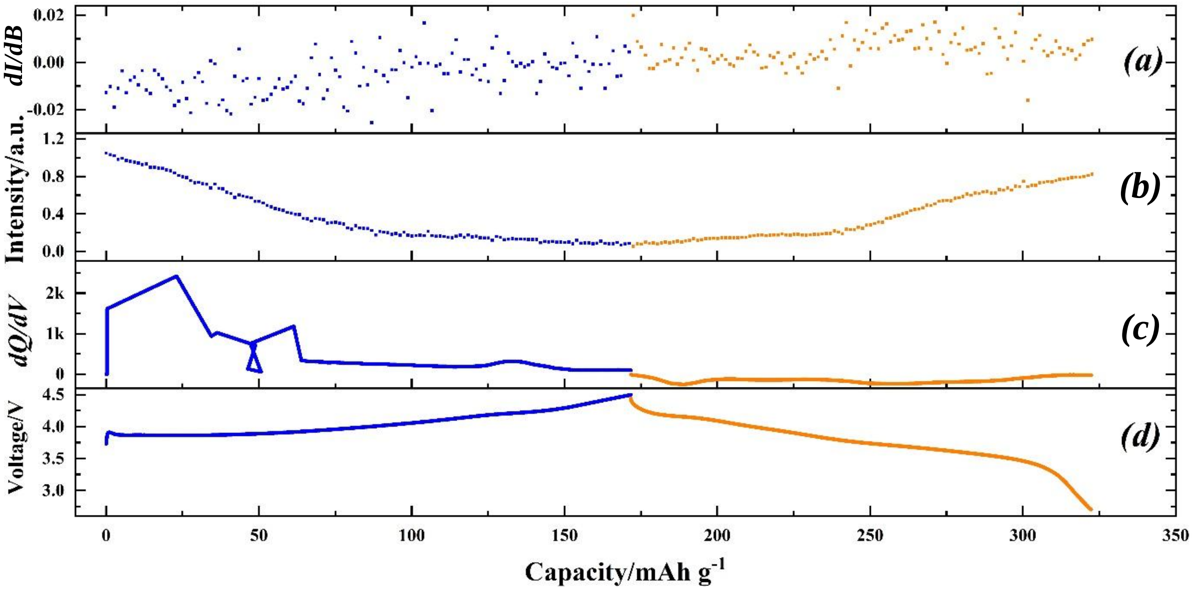

Figure S5. (a) The first derivative of (b) the EPR signal intensity of NMC811 (broad signal); (c) dQ/dV and (d) the voltage change of the NMC811 cathode during charge (blue) and the discharge (orange) process in LP57 without VC additive at a C rate of 1/10C from 2.7 V to 4.5V during its first cycle.

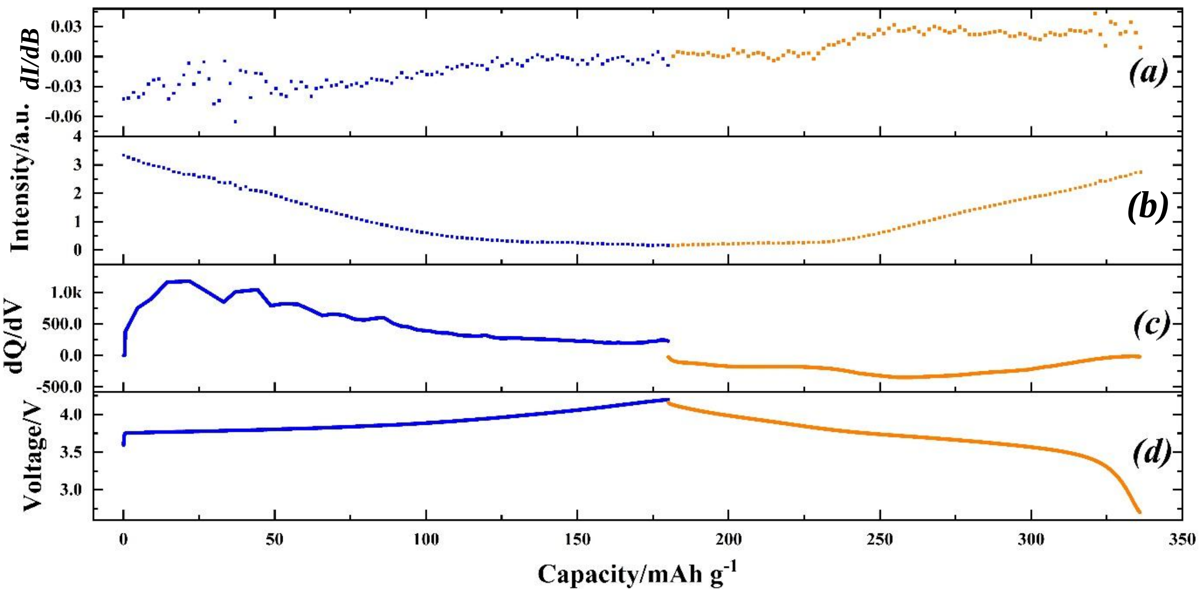

Figure S6. (a) The first derivative of (b) the EPR signal intensity of NMC811 (broad signal); (c) dQ/dV and (d) the voltage change of the NMC811 cathode during charge (blue) and the discharge (orange) process in LP57 with 2% VC additive at a C rate of 1/10C from 2.7 V to 4.5V during its first cycle.

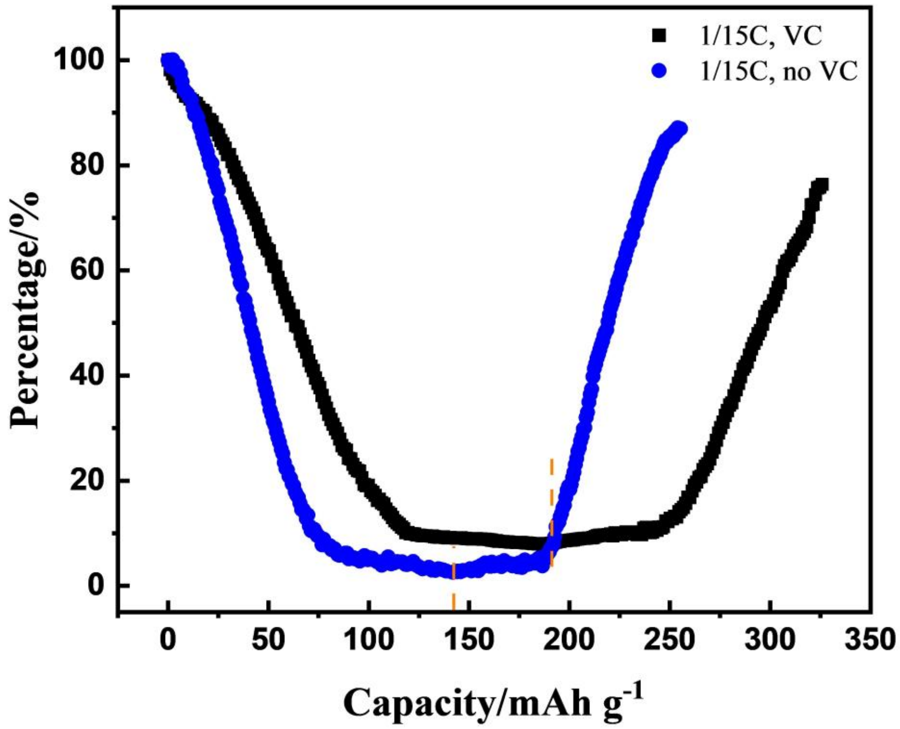

.

Figure S7. Normalized EPR signal intensity of NMC811 under different SOC in LP57 with and without VC additive after 100 cycles.
